# The impact of national comprehensive medical reform on residents' medical expenses: Evidence from China

**DOI:** 10.3389/fpubh.2022.1038543

**Published:** 2023-01-05

**Authors:** Changfei Nie, Yuan Feng

**Affiliations:** ^1^School of Economics and Management, Nanchang University, Nanchang, China; ^2^College of City Construction, Jiangxi Normal University, Nanchang, China

**Keywords:** national comprehensive medical reform, pilot policy, medical expenses, China, difference-in-differences

## Abstract

Residents' high medical expenses is the core challenge that needs to be solved urgently in China's medical reform for a long time. Based on the panel data of 30 provinces in Chinese Mainland during 2011–2019, we evaluate the impact of China's national comprehensive medical reform pilot policy on residents' medical expenses by using the difference-in-differences model. The results show that the pilot policy was generally conducive to reducing residents' medical expenses, resulting in a reduction of 2.13% in per capita medical expenses for inpatients, but the effect on per capita medical expenses for outpatients was insignificant. Mechanism analysis shows that hospital competition and institutional environment played a moderating role in the effect of the pilot policy on residents' medical expenses reduction. The more intense the hospital competition and the better the institutional environment, the more significant of the reduction effect. In addition, the reduction effect of the pilot policy was greater in the central provinces, the provinces with poor medical infrastructure, and the provinces with strong financial strength. This study provides useful policy insights for deepening medical reform and reducing residents' medical expenses.

## Introduction

The rapid rise of medical expenses will crowd out residents' consumption in other fields, bring about a loss of social welfare, and thus affecting the sustainable development of macro-economy (1, 2). Therefore, how to effectively cope with the continuous rising in residents' medical expenses has become a common challenge for the medical systems worldwide, which is also a problem that national medical reform hope to solve (3). For example, Germany, the United Kingdom and the United States have made efforts to reduce residents' medical expenses by establishing social medical insurance system, national medical service system and commercial medical insurance system, respectively.

As the largest developing country in the world, China realized the incremental expansion of medical and health service supply since the market-oriented reform of medical and health services in the late 1990s. It greatly contributed to the rapid development of the medical service industry (4). However, due to the concentration of medical and health resources in large cities, medical institutions tend to choose “big prescriptions” or “high priced drugs,” which made “expensive medical expenses” became a more prominent economic and social problem (5). In China, poverty caused by high medical expenses accounts for 42.3% of the total number of people in poverty (6). The high medical expenses seriously restrict people's demand for a good and healthy life.

**Figure 1:**
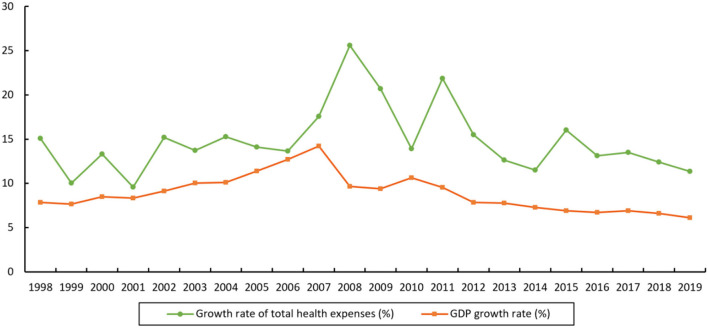
Growth rate of total medical expenses and GDP growth rate in China during 1998-2019. Data sources: China Health and Health Statistical Yearbook, China Statistical Yearbook.

Figure 1 shows the changing trend of growth rate of total medical expenses and GDP in China during 1998–2019. Since 1998, China's total medical expenses have maintained a rapid annual growth rate of 14.8%, which is 1.7 times than the GDP growth rate in the same period. Since 2011, the gap between these two growth rates has even reached nearly two times. Moreover, with the deepening of population aging, medical expenses are expected to keep rising.

In order to cope with the rapid rising trend of medical expenses, the Chinese government has implemented a series of medical and health system reforms. In 1999, China began to establish a medical insurance system for urban workers. In March 2009, the Central Committee and the State Council of Communist Party of China (CPC) issued “*the Opinions on Deepening the Reform of the Medical and Health System*.” The “New Medical Reform” thereafter beginning, which aims to effectively reduce the burden of residents' medical expenses (7, 8). On this basis, in order to explore a new path for the medical and health system reform, the leading group for medical reform of the State Council carried out two batches of national comprehensive medical reform (NCMR) pilot provinces and identified a total of 11 pilot provinces in January 2015 and May 2016, expecting to further reduce residents' medical expenses.

Against this contextual backdrop, we use the exogenous shock of China's NCMR pilot policy, and build a difference-in-differences (DID) model to test the impact of the pilot policy on residents' medical expenses. Compared with existing literature, the marginal contributions of this study are mainly in the following three aspects. First, in terms of research topics, although the NCMR pilot policy has been implemented for more than seven years, there was little literature on the quantitative evaluation of the policy's implementation effects. Although a few studies focused on the policy (9), they mainly used the qualitative research methods such as case study rather than quantitative empirical analysis, making it difficult to evaluate the policy effect comprehensively and accurately. Based on the analysis framework of DID method, this study evaluates the net effect of the pilot policy on residents' medical expenses. We further ensure the robustness of the research conclusion through a series of robustness tests such as the parallel trend test, the propensity score matching (PSM) and DID method (PSM-DID) estimation, the entropy balancing estimation, the placebo test, controlling the time trend, and changing the sample interval. Second, in terms of research contents, this study not only examines the impact of the NCMR pilot policy on residents' medical expenses, but also further explores the mechanisms of which the pilot policy affects medical expenses from the micro and macro perspectives. Additionally, we also examine the heterogeneous impact of the pilot policy on residents' medical expenses from the perspectives of region, medical infrastructure, and financial strength. Third, in terms of practical sense, this study provides the latest empirical evidence of the NCMR pilot policy to reduce residents' medical expenses. The relevant research conclusions are helpful to accurately understand and grasp the achievements and shortcomings of the reform, which provide references for further promoting the medical reform and reducing residents' medical expenses.

The remainder of this study is organized as follows: the second section is the literature review, the third section introduces the methods and data, the fourth section is empirical results and analyses, the fifth section is further analyses, the sixth section is discussion, and the seventh section is conclusion.

## Literature review

The rapid rise of residents' medical expenses is a common problem in medical reform around the world, and also a hot topic in academic research. In general, the existing literature comprehensively investigated the influencing factors of residents' medical expenses from multiple perspectives. Some studies believed that serious air pollution is not conducive to residents' health, which may increase residents' medical expenses (10–14), while strict environmental regulations can alleviate this adverse effect to a certain extent (15, 16). Other studies explained residents' high medical expenses from the perspectives of fiscal decentralization (17) and energy use (18).

In addition, many literatures discussed the impact of medical reform on residents' medical expenses. Kuroki (19) used the implementation of the United States Tax Cuts and Jobs Act in 2017 as a shock to examine the impact of health insurance on residents' out-of-pocket medical expenses, and found that the new law increased the tax rate of medical expenses deduction and the total amount of medical expenses deduction, but the increase was greater in states with large uninsured population. Vuong (20) argued that the amended Law on Health Insurance in Vietnam may lead to risk of destitution of the poor. Through a cross-sectional study, Albright et al. (21) explored the effect of The Patient Protection and Affordable Care Act on residents' out-of-pocket cost burden and found that the reform only had small improvements. Kobayashi et al. (22) investigated the impact of a Japanese fracture liaison service on medical expenses of patients, and found that the service can reduce overall healthcare costs.

As for China, many scholars also paid attention to this topic. Based on the China Health and Retirement Longitudinal Study (CHARLS) database in 2013, 2015 and 2018, Liu and Hu (23) assessed the impact of the long-term care policy pilot on residents' medical expenses and found that the policy significantly reduced the outpatient, inpatient and average daily hospitalization expenses of the elderly. Using the medical expenses data of internal and surgical inpatients of a hospital in Beijing from June 2016 to December 2019, Xiao et al. (24) conducted a regression discontinuity design (RDD) to test the impact of the comprehensive reform of separation of medicine and pharmacy and the comprehensive reform of medical consumption linkage on residents' medical expenses, and the results showed that the two reforms significantly reduced the proportion of drugs and medical consumption. Liu et al. (25) used the monthly data from 2013 to 2019 to evaluate the impact of China's medical pricing reform on the medical expenses of outpatients and inpatients, and found that this policy generally reduced the expenses of two types of patients. Zhang et al. (26) regarded the price reform of drugs and medical services in public hospitals implemented in China in 2015 as a quasi-natural experiment, and used the DID model to test the effect of the policy, finding that the policy was effective in reducing drug costs but increased the cost of inpatient surgery for residents by 9.1%. Liu et al. (27) evaluated the impact of the New Rural Cooperative Medical System on the medical expenses of rural elderly chronic disease patients, and found that the policy significantly reduced the medical expenses of outpatients, but not reduced the medical expenses of inpatients.

Throughout the existing literature, we can find that the existing literature comprehensively analyzed the effects of many medical reform policies. However, China's NCMR pilot policy has received less attention. Wu et al. (9) took Zhejiang Province as an example, and analyzed the effect of China's NCMR pilot policy by using the case study method, finding that the policy achieved remarkable results in management, financing and fair investment, resource creation, service provision and health care. By comparing the relevant data of the pilot province of Jiangsu and the non-pilot province of Shandong before and after China's NCMR pilot policy implementation, Wang (28) found that the pilot policy effectively reduced residents' medical expenses. Unlike the above literature, which mainly focused on China's NCMR pilot policy effect in one pilot province, this study evaluates the policy effect of all 11 pilot provinces, enabling a more comprehensive and in-depth understanding of the effects and shortcomings of the policy. This can make a powerful supplement to the existing literature.

## Policy background and hypotheses

### Policy background

The pilot governance is a typical governance mode with Chinese characteristics, which was widely used in economy, ecology, public service and other reform fields (29). Since the Chinese government implemented the “New Medical Reform” in 2009, residents' medical expenses have not been substantially reduced, and the problem of “expensive medical expenses” is still prominent (30). In this context, in order to explore an effective way to reduce residents' medical expenses, the leading group for medical reform of the State Council started the pilot work of NCMR in some provinces under the guidance of the “early and pilot progressive reform strategy,” with a view to “wade out a new path” in the work of medical reform. In January 2015, Jiangsu, Anhui, Fujian and Qinghai were identified as the first batch of NCMR pilot provinces. Further, seven provinces including Shanghai and Zhejiang were identified as the second batch of NCMR pilot provinces in May 2016, marking the continuous advancement of NCMR. In practice, in order to effectively promote the pilot work, the pilot provinces successively issued more specific implementation plans, which carried out many beneficial explorations around the core issues such as reducing residents' medical expenses. It can be seen that the implementation of the NCMR pilot policy effectively expanded the depth and breadth of the “New Medical Reform,” and also provided an opportunity for this study to comprehensively evaluate the policy effect. Table 1 shows China's NCMR pilot provinces.

**Table 1:** China's NCMR pilot provinces.

**Year**	**NCMR pilot provinces**
2015	Jiangsu, Anhui, Fujian, Qinghai
2016	Shanghai, Zhejiang, Hunan, Chongqing, Sichuan, Shaanxi, Ningxia

The table is collected and created by this study.

### Hypotheses

**Figure 2:**
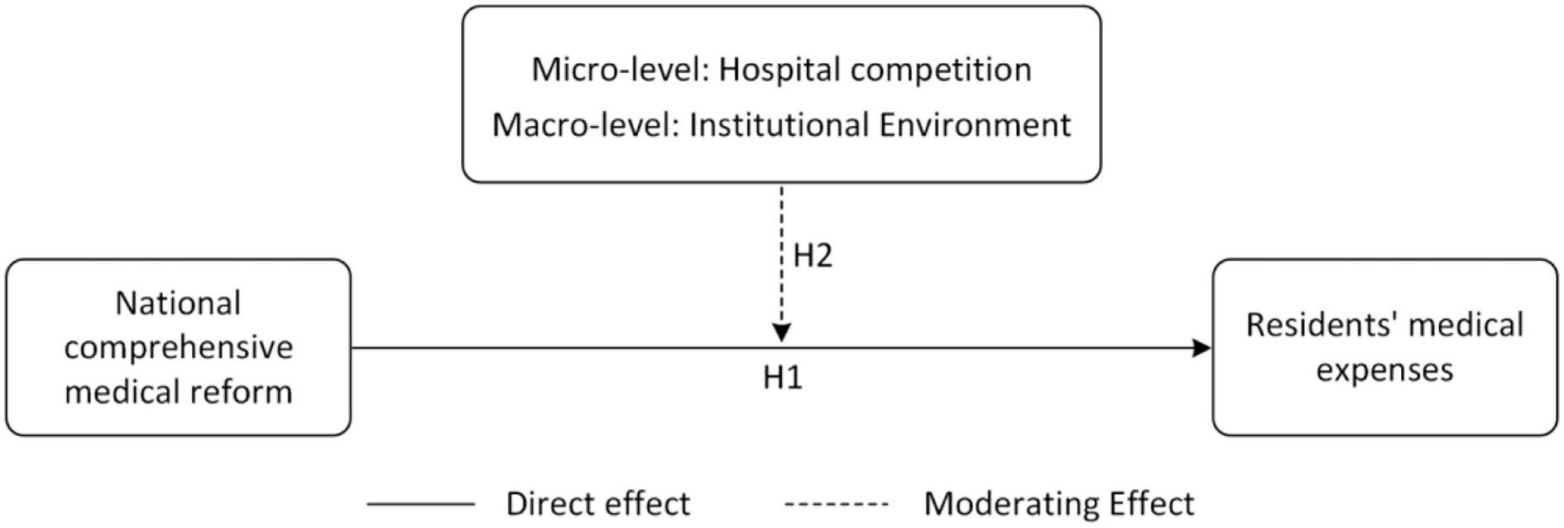
Research framework.

We will comprehensively investigate the impact of China's NCMR pilot policy on residents' medical expenses from two aspects: the direct effect and the moderating effect. Among them, the analysis of the moderating effect will be carried out from both the micro and the macro levels. Figure 2 shows the research framework of this study.

#### The direct effect

The problem of “expensive medical expenses” is the primary issue that China's NCMR pilot policy striving to address. In practice, the pilot provinces have adhered to the basic principles of public welfare, full coverage and synergy, and also innovated a number of policy measures to reduce residents' medical expenses (28). First, in terms of public welfare, the comprehensively reform of public hospitals was carried out to break the practice of “subsidizing doctors with drugs.” For example, in the process of implementing the pilot policy, Jiangsu Province realized zero margin sales of drugs by increasing government investment in public hospitals. Second, in terms of full coverage, the medical insurance system was optimized to improve the scope of benefits of the medical service security system. For instance, by integrating the basic medical insurance system for urban and rural residents, Anhui Province has promoted the expansion and upgrading of basic medical insurance. Third, in terms of synergy, during the implementation of the pilot policy, special emphasis was placed on taking multiple measures to give play to the policy overlap effect. For this reason, the following hypothesis can be proposed as:

**Hypothesis 1:** *China's NCMR pilot policy was conducive to reducing residents' medical expenses*.

#### The moderating effect

In theory, whether the effect of China's NCMR pilot policy to reduce residents' medical expenses can be fully realized is affected by many factors at the micro and macro levels.

On the one hand, at the micro-level, hospital competition is an important factor. For a long time, China's access to the medical service market is relatively strict, adhering to the policy of public institutions taking the lead and private institutions as the supplement (31). As a result, public hospitals occupy a dominant position in China's medical service system, and residents mainly rely on public hospitals when seeking medical treatment. At the same time, the salary system of medical staff in public hospitals usually adopts the combination of fixed salary and performance salary, and performance salary is usually closely related to medical and pharmaceutical income, which leads to the more serious phenomenon of “subsidizing doctors with drugs” (32). In this context, liberalizing hospital market access, developing private hospitals, introducing stronger competition on the medical supply side, and introducing competition mechanism to solve the problem of excessive medical treatment associated with monopoly are considered to be effective tools to control the rise of medical expenses (33). Therefore, in the provinces where the number of private hospitals is relatively large and the competition between hospitals is more intense, the pilot policy can reduce residents' medical expenses more effectively.

On the other hand, at the macro-level, institutional environment is also an important factor. Market-oriented reform is an important direction of China's NCMR pilot policy (28, 30). For example, in the process of policy implementation, Anhui Province, Jiangsu Province and other provinces have particularly emphasized the role of market mechanism in the formation of medical prices and the reform of the salary system. However, whether the idea of market-oriented reform can be effectively implemented depends largely on the influence of local institutional environment. Only in provinces where the institutional environment is relatively good and the government's intervention is relatively weak can the relevant measures of the pilot policy be fully implemented. On the contrary, in provinces with poor institutional environment and strong government intervention, the relevant measures of the pilot policy may encounter many obstacles, or even struggle. Therefore, the quality of the institutional environment also plays an important moderating role in the process of reducing residents' medical expenses through the pilot policy. For this reason, the following hypothesis can be proposed as:

**Hypothesis 2:** *The effect of China's NCMR pilot policy on residents' medical expenses reduction was affected by hospital competition and institutional environment. The more intense the hospital competition and the better the institutional environment, the more significant the effect of the pilot policy on residents' medical expenses reduction*.

## Methods and data

### Benchmark model construction

Based on the analysis framework of DID, we examine the impact of the NCMR on residents' medical expenses. The benchmark model is constructed as follows:


(1) $$\begin{array}{l} \ln\;ME_{it}=\beta_{0}+\beta_{1}Policy_{it}+\varphi X_{it}+\mu_{i}+\eta_{t}+\varepsilon_{it} \end{array}$$

Where the subscript *i* and *t* represent the province and year, ln*ME* is the explained variable, indicating residents' medical expenses; *Policy* is the core explanatory variable, referring to China's NCMR pilot policy; *X* denotes a series of control variables that may affect residents' medical expenses. In the benchmark model, we also control for the province fixed effects (*Province FE*) μ_*i*_ and the year fixed effects (*Year FE*) η_*t*_to capture the unobservable factors of provinces and years. ε_*it*_ represents the error term.

Our main concern is the regression coefficient β_1_ of *Policy*. A significantly negative β_1_ indicates that China's NCMR pilot policy is conducive to reducing residents' medical expenses. On the contrary, if β_1_ is positive significant or insignificant, it indicates that the pilot policy fails to effectively solve the problem of “expensive medical expenses.”

### Moderating effect model construction

In order to explore the mechanisms of China's NCMR pilot policy affecting residents' medical expenses, we further construct the following moderating effect model (34):


(2) $$\begin{array}{l} \ln\;ME_{it}=\alpha_{0}+\alpha_{1}Policy_{it}+\alpha_{2}\times \left(Policy_{it}\times M_{it}\right)+\alpha_{3}M_{it}\\ +\varphi X_{it}+\mu_{i}+\eta_{t}+\varepsilon_{it} \end{array}$$

Where, *M* represents the moderating variables. Corresponding to **Hypothesis 2**, we construct the moderating variables from both the micro and the macro levels, and the definitions of other variables in this model are consistent with the benchmark model.

In model (2), we mainly focus on the regression coefficient α_2_ of the interaction term *Policy* × *M*, a significant α_2_ indicates that the moderating variables do play a moderating role in the process of China's NCMR pilot policy affecting residents' medical expenses.

### Variable definitions

#### Explained variables

We use per capita medical expenses to measure the residents' medical expenses, which is specifically divided into two categories: outpatients and inpatients. To ensure that medical expenses are comparable across years, we convert the per capita medical expenses to constant 2011 prices. On this basis, we take logarithm of the per capita medical expenses to mitigate the effect of heteroskedasticity. Therefore, there are two explained variables in this study: (1) ln*ME_outpatient* represents the logarithm of per capita medical expenses of outpatients, and (2) ln*ME_inpatient* denotes the logarithm of per capita medical expenses of inpatients.

#### Core explanatory variable

We take China's NCMR pilot policy as a quasi-natural experiment and use the dummy variable to reflect the pilot policy. Specifically, if province *i* was identified as the pilot in year *t*, the value of *Policy*_*it*_ in and after year *t* is set to 1, otherwise the value is set to 0. In 2015 and 2016, four and seven provinces were selected as pilot provinces, respectively, constituting the experimental group, and the remaining 19 provinces constitute the control group.

#### Moderating variables

According to **Hypothesis 2**, we construct the moderating variables from both the micro and the macro levels.

(1) Hospital competition (*Competition*). Referring to the existing research (35), we use the proportion of private hospitals to the total number of hospitals to reflect the hospital competition. The higher the value, the more intense the competition among hospitals.
(2) Institutional environment (*Market*). We apply the *Marketization Index* to measure the institutional environment, which is widely used in existing literature (17, 36). The larger the *Marketization Index*, the better the institutional environment.

#### Control variables

To avoid the possible influence of other random factors on the regression results, we further control for the following variables:

(1) Economic development level (ln*Pgdp*). Economic development level is an important factor affects residents' medical expenses (10, 35). Therefore, we take the actual per capita GDP as a control variable. The per capita GDP is converted into the constant price in 2011.
(2) Industry structure (*Indus*). Industry structure affects the quality and level of healthcare services (17), which may further have an impact on residents' medical expenses. Based on this consideration, we incorporate industrial structure as a control variable, which is expressed by the ratio of the added value of the secondary and tertiary industries to GDP.
(3) Residents' health status (*Health*). Residents' health status is another important factor that affects medical expenses (37). Drawing on the existing literature (17), we choose the mortality rate as the measurement variable of residents' health status.
(4) Environmental pollution (ln*Pollution*). Numerous studies showed that severe air pollution can cause various diseases and affect residents' physical and mental health (10, 38). Therefore, we incorporate air pollution as a control variable in the benchmark model, which is expressed by the logarithm of per capita industrial sulfur dioxide emissions (17).
(5) Ecological endowment (*Forest*). Ecological endowment may indirectly affect residents' medical expenses by affecting their health. Therefore, we also introduce this as a control variable, which is measured by forest coverage (10).
(6) Medical support (*Health-expenses*). The total health expense is an important variable reflecting medical support of local governments and has a significant impact on residents' medical expenses. In this study, we use the logarithm of the total health expenses per capita to reflect medical support.
(7) Medical infrastructure (*Hospital-bed*). Medical infrastructure is another important factor that determines residents' medical expenditure (17). Based on the existing research (17), we use the number of hospital beds per capita to measure.

### Data and descriptive statistics

We use the panel data of 30 provinces (excluding Tibet) in Chinese Mainland during 2011–2019 as the research sample to evaluate the net effect of China's NCMR pilot policy on residents' medical expenses. The data are mainly obtained from the *China Health and Family Planning Statistical Yearbook*, the *China Health and Health Statistical Yearbook*, the *China Forestry Statistical Yearbook*, and the *China Statistical Yearbook*. Descriptive statistics of relevant data are shown in Table 2.

**Table 2:** Descriptive statistics.

**Variable type**	**Variables**	**Obs**	**Mean**	**S.D**.	**Min**	**Max**
Explained variables	ln*ME_outpatient*	270	0.7622	0.2631	0.0995	1.7026
	ln*ME_inpatient*	270	4.3654	0.3231	3.7370	5.4309
Core explanatory variable	*Policy*	270	0.1778	0.3830	0.0000	1.0000
Moderating variables	*Competition*	270	0.5053	0.1438	0.1221	0.8039
	*Market*	270	6.7254	1.9778	2.3300	11.4000
Control variables	ln*Pgdp*	270	10.8164	0.4350	9.7058	11.8286
	*Indus*	270	90.2628	5.1196	73.8000	99.7000
	*Health*	270	6.0876	0.7821	4.2600	7.5700
	ln*Pollution*	270	4.2227	1.1256	−0.9117	6.3945
	*Forest*	270	32.9864	17.8433	4.0200	66.8000
	*Health-expenses*	270	0.3346	0.1692	0.1200	1.3538
	*Hospital-bed*	270	51.5847	10.4454	30.2192	80.6682

## Empirical results and analyses

### Benchmark regression

The benchmark regression results are reported in Table 3. Column (1) shows that the estimated coefficient of *Policy* is 0.0044 but not significant, indicating that China's NCMR pilot policy had no significant impact on residents' medical expenses of outpatients. Column (2) presents that the estimated coefficient of *Policy* is−0.0213 and significant at the 1% level, indicating that the pilot policy significantly reduced residents' medical expenses of inpatients. In terms of economic significance, the pilot policy resulted in a reduction of approximately 2.13% in per capita medical expenses of inpatients. Since residents' medical expenses for inpatients is much higher than that for outpatients (see Table 2), in general, the comprehensive medical reform solved the problem of “expensive medical expenses” to a certain extent, which means that the reform was effective.

**Table 3:** Benchmark regression results.

**Variables**	**(1) ln*ME_outpatient***	**(2) ln*ME_inpatient***
*Policy*	0.0044	−0.0213^***^
	(0.0086)	(0.0080)
ln*Pgdp*	0.3366^***^	0.1146
	(0.0825)	(0.0763)
*Indus*	0.0047^**^	0.0071^***^
	(0.0023)	(0.0021)
*Health*	0.0150^*^	−0.0101
	(0.0084)	(0.0078)
ln*Pollution*	0.0147	−0.0075
	(0.0094)	(0.0087)
*Forest*	−0.0043^**^	−0.0028
	(0.0020)	(0.0019)
*Health-expenses*	0.1137	−0.0212
	(0.0707)	(0.0654)
*Hospital-bed*	−0.0023^**^	−0.0021^**^
	(0.0010)	(0.0009)
*Constant*	−3.4547^***^	2.5557^***^
	(0.8207)	(0.7588)
*Province FE*	Yes	Yes
*Year FE*	Yes	Yes
*N*	270	270
*R* ^2^	0.9706	0.9601

Standard errors are in parentheses. ^***^, ^**^, and ^*^ indicate significance at the 1, 5, and 10% levels, respectively.

### Robustness tests

#### Parallel trend test

The applicability of the DID model is based on the satisfaction of the parallel trend assumption. In our study, it is required that before the implementation of China's NCMR pilot policy, there is no systematic difference in the change trend of per capita medical expenses between the experimental group and the control group. Therefore, we refer to the existing literature and use the event study method to verify the parallel trend (39). The specific model is constructed as follows:


(3) $$\begin{array}{c} \ln\;ME_{it}=\alpha_{0}+\overset{-2}{\underset{t=-5}{\sum }}\beta_{t}\times before_{it}+\overset{4}{\underset{t=0}{\sum }}\beta_{t}\times after_{it}+\varphi X_{it}\\ +\mu_{i}+\eta_{t}+\varepsilon_{it} \end{array}$$

Where *before*_*it*_ and *after*_*it*_ are the core explanatory variables, representing the dummy variables from the implementation year of the pilot policy. Their value rules are: if the experimental group are in the *t* year before (after) being included in the pilot scope, then *before*_*it*_ (*after*_*it*_) takes the value of 1, and the other cases take the value of 0. In the empirical analysis, we use the year before implementation (*t* = −1) as the comparison period to avoid the influence of multiple collinearities.

**Figure 3:**
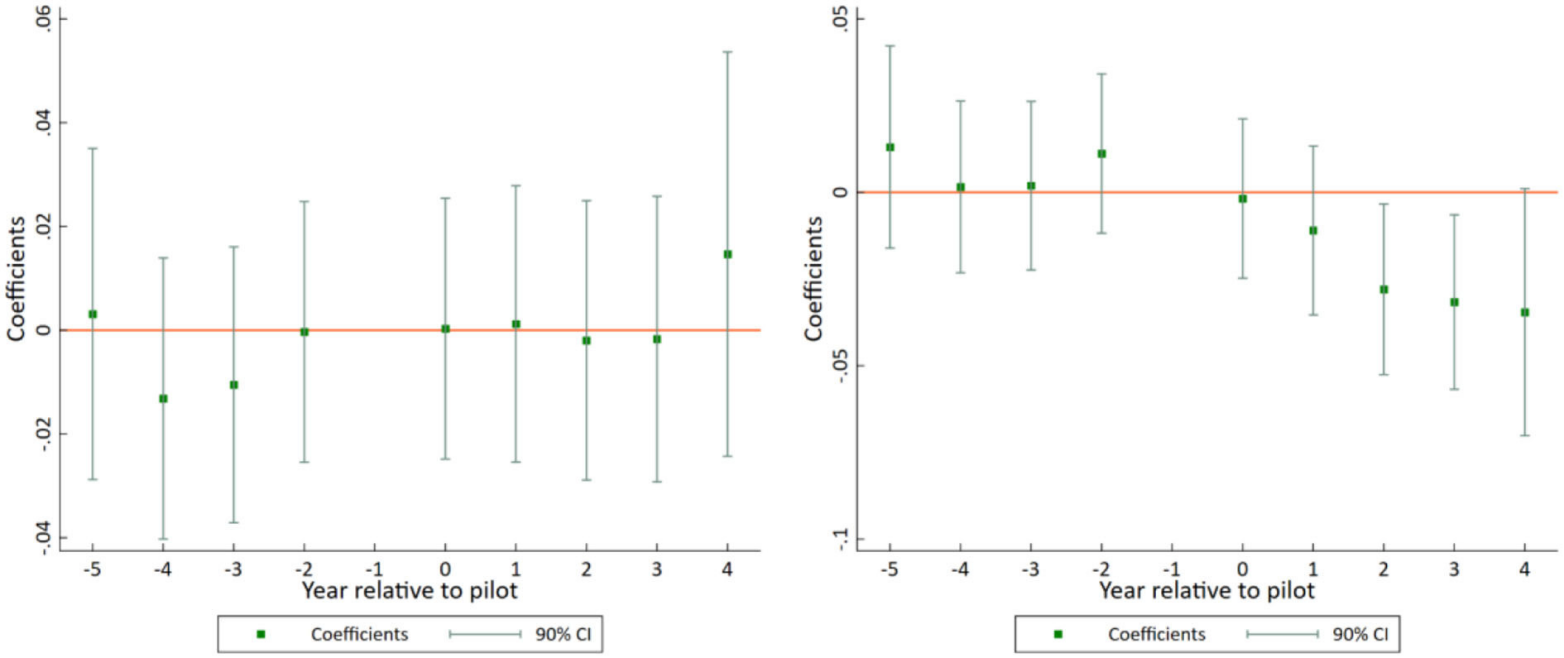
Parallel trend test of ln*ME_outpatient* **(left)** and ln*ME_inpatient* **(right)**.

Figure 3 plots the regression results according to Equation (3). As can be seen, whether ln*ME_outpatient* or ln*ME_inpatient* is the explained variable, the estimated coefficient of *before*_*it*_ is not significant, indicating that there is no systematic difference in the change trend of the per capita medical expenses between the experimental and control group samples before the implementation of China's NCMR pilot policy. Therefore, the parallel trend hypothesis is verified, and they DID model adopted in this study is reasonable.

Meanwhile, when ln*ME_outpatient* is used as the explained variable, none of the estimated coefficients of *after*_*it*_ are significant, indicating that there is no significant effect of the pilot policy on residents' medical expenses of outpatients. When ln*ME_inpatient* is used as the explained variable, the estimated coefficients of *after*_*it*_ are significantly negative at 2 and 3 years after the policy implementation (*t* = 2 & *t* = 3), indicating that there is a significant negative effect of the pilot policy on residents' medical expenses of inpatients, which is consistent with the results of the benchmark regression.

#### PSM-DID estimation

**Figure 4:**
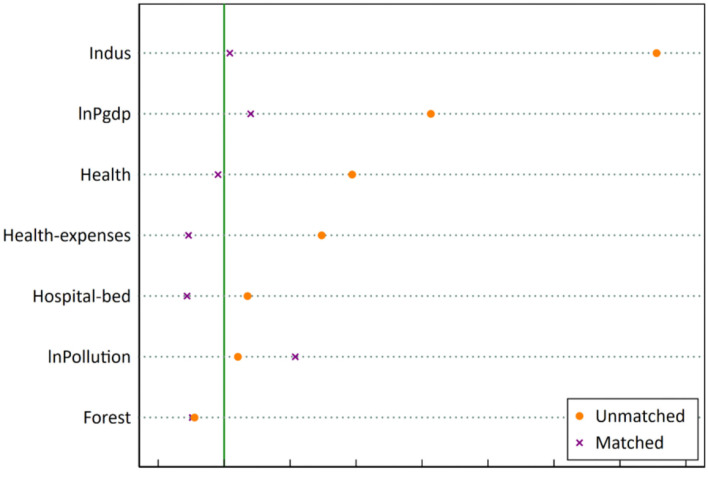
PSM balance test results.

Considering that the identification of experimental group provinces may not be random, it may lead to the problem of “selection bias” in the model estimation process. In order to overcome this issue, we further employ the PSM-DID to conduct the basic regression. Specifically, we choose the explained variables in the benchmark model as outcome variables and the seven control variables as the covariates, and use the kernel matching method to match the experimental and control groups. The PSM balance test results are shown in Figure 4. It can be seen that among the covariates, the absolute value of the standardized bias of all other covariates do not exceed 10%, except for environmental pollution (ln*Pollution*), which reaches 10.8%. At the same time, the mean value difference of all covariates after matching is not significant, indicating that the experimental and the control group are very close. Therefore, the matching result is good.

Based on the matched samples, the DID estimation is performed again based on Equation (1), and the regression results are shown in Table 4. It can be seen that the coefficient of *Policy* in column (1) is−0.004 but insignificant, while the coefficient of *Policy* in column (2) is−0.0237 and significant at the level of 1%, which further supports the benchmark regression results.

**Table 4:** PSM-DID estimation.

**Variables**	**(1) ln*ME_outpatient***	**(2) ln*ME_inpatient***
Policy	−0.0004	−0.0237^***^
	(0.0098)	(0.0086)
Control variables	Yes	Yes
Constant	Yes	Yes
Province FE	Yes	Yes
Year FE	Yes	Yes
*N*	226	226
*R* ^2^	0.9680	0.9600

Standard errors are in parentheses. ^***^, ^**^, and ^*^ indicate significance at the 1, 5, and 10% levels, respectively.

#### Entropy balancing estimation

Compared with the PSM method, the entropy balancing method can achieve sample balance of the control and experimental group in a higher dimension without causing sample loss (40). Therefore, we use the entropy balancing method to re-examine. The steps of entropy balancing method are as follows: First, taking the control variables of the benchmark model as the characteristic variables. Second, obtaining a set of weights so that the mean, variance, and skewness of the characteristic variables in the experimental and control provinces are approximately equal. Third, using the weights for weighted least squares (WLS) estimation. The estimation results of the entropy balancing estimation are shown in Table 5. We can see that the coefficient of *Policy* in column (1) is−0.0178 and insignificant, and the coefficient of *Policy* in column (2) is significantly negative, which demonstrates the robustness of the core research findings again.

**Table 5:** Entropy balancing estimation.

**Variables**	**(1) ln*ME_outpatient***	**(2) ln*ME_inpatient***
Policy	−0.0178	−0.0529^***^
	(0.0119)	(0.0133)
Control variables	Yes	Yes
Constant	Yes	Yes
Province FE	Yes	Yes
Year FE	Yes	Yes
*N*	270	270
*R^2^*	0.9880	0.9933

Standard errors are in parentheses. ^***^, ^**^, and ^*^ indicate significance at the 1, 5, and 10% levels, respectively.

#### Placebo test

**Figure 5:**
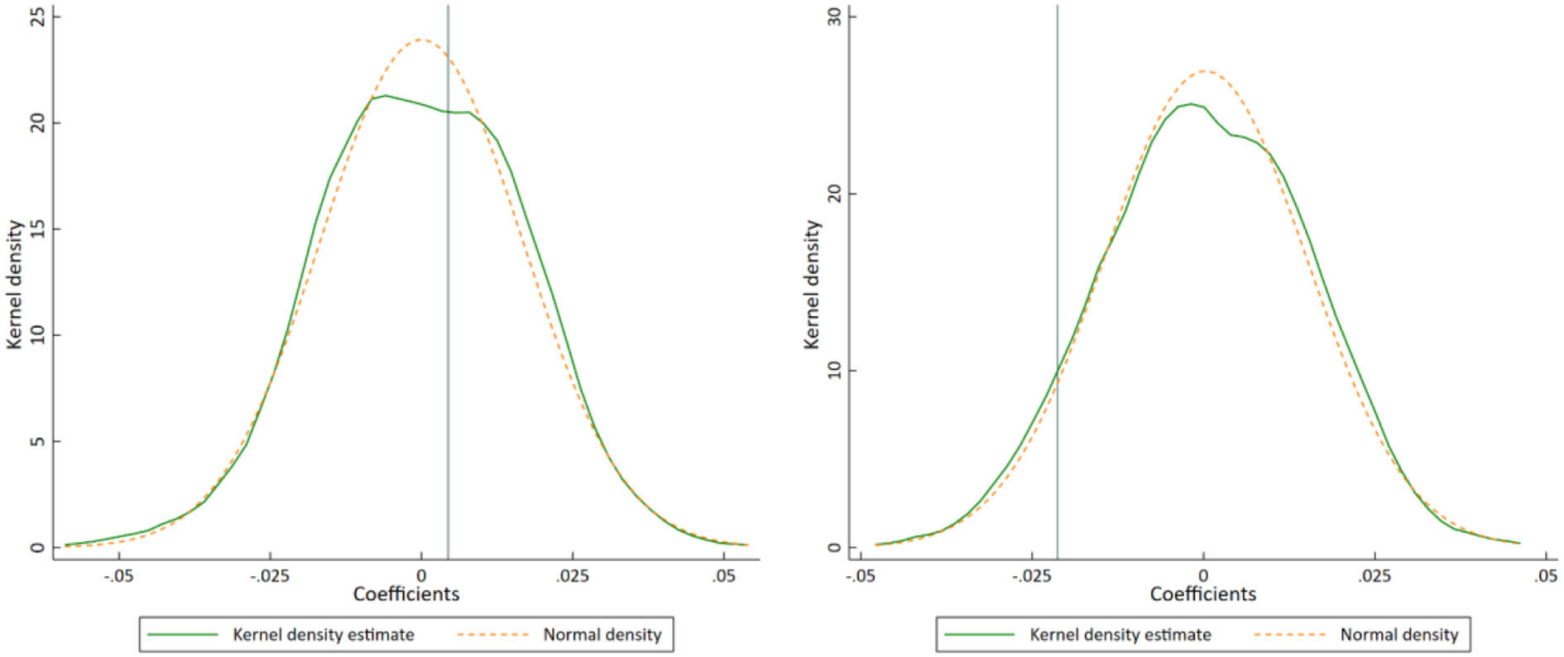
Placebo test of ln*ME_outpatient* **(left)** and ln*ME_inpatient* **(right)**. Notes: The teal vertical lines represent the coefficients of the Benchmark regression in Table 3.

In order to exclude the interference of other random factors on the regression results, we further conduct a placebo test for robustness. Specifically, among the 30 provinces, we randomly select four provinces as the pilot provinces in 2015 and other seven provinces as the pilot provinces in 2016, and construct a virtual policy variable for regression. We repeat the above process for 1,000 times to obtain 1,000 “spurious” regression coefficients of *Policy*, and plot the kernel density curve (see Figure 5). According to the statistics, when ln*ME_outpatient* is used as the explained variable, 415 regression coefficients are larger than the benchmark regression coefficient of *Policy* (0.0044), when ln*ME_inpatient* is taken as the explained variable, only 75 coefficients are smaller than the benchmark regression coefficient (-0.0213), and none of the regression coefficients of *Policy* is significant, indicating that the pilot policy has no significant impact on the medical expenses of outpatients, but it can significantly reduce the medical expenses of inpatients.

#### Control time trend

Random factors such as the time trend of the control variables may also have an impact on the regression results (41). Therefore, we further incorporate the interaction term between the control variables and the time trend term on the basis of the benchmark model and re-estimate it. The regression results are shown in Table 6. It can be seen that the effect of China's NCMR pilot policy on the medical expenses of outpatients is not significant, and the effect on the medical expenses of inpatients is significantly negative, which is consistent with the benchmark regression results.

**Table 6:** Control time trend.

**Variables**	**(1) ln*ME_ outpatient***	**(2) ln*ME_ inpatient***
Policy	0.0125	−0.0205^**^
	(0.0085)	(0.0085)
Control variables	Yes	Yes
T × control variables	Yes	Yes
Constant	Yes	Yes
Province FE	Yes	Yes
Year FE	Yes	Yes
*N*	270	270
*R^2^*	0.9759	0.9619

Standard errors are in parentheses. ^***^, ^**^, and ^*^ indicate significance at the 1, 5, and 10% levels, respectively.

#### Change sample interval

The selection of the sample interval may also have an impact on the empirical results (39). For this reason, we use the research samples during 2012–2018 and 2013–2017 for regression, and the results are reported in Table 7. It can be seen that the research conclusion of benchmark regression remains unchanged.

**Table 7:** Change sample interval.

**Variables**	**2012–2018**		**2013–2017**	
	**(1) ln** ***ME_ outpatient***	**(2) ln** ***ME_ inpatient***	**(3) ln** ***ME_ outpatient***	**(4) ln** ***ME_ inpatient***
Policy	0.0102	−0.0170^**^	0.0087	−0.0127^*^
	(0.0085)	(0.0079)	(0.0077)	(0.0076)
Control variables	Yes	Yes	Yes	Yes
Constant	Yes	Yes	Yes	Yes
Province FE	Yes	Yes	Yes	Yes
Year FE	Yes	Yes	Yes	Yes
*N*	210	210	150	150
*R^2^*	0.9612	0.9454	0.9544	0.9273

Standard errors are in parentheses. ^***^, ^**^, and ^*^ indicate significance at the 1, 5, and 10% levels, respectively.

## Further analyses

### Mechanism analysis

In order to further explore the mechanisms of China's NCMR pilot policy to reduce residents' medical expenses, we estimate the moderating effect model of Equation (2). The regression results are shown in Table 8. As can be seen in columns (1) and (2), when *Competition* is used as the moderating variable, the interaction item *Policy* × *Competition* are all significantly negative, indicating that hospital competition does play a moderating role. Specifically, the more intense the competition among hospitals (i.e., the higher the ratio of private hospitals), the stronger the reduction effect of China's NCMR pilot policy on residents' medical expenses. As can be seen in columns (3) and (4), when *Market* is used as the moderating variable, the interaction item *Policy* × *Market* are all negative, and column (3) is significantly negative at the 1% level, which partly reflects the moderating role of institutional environment. Specifically, with a better institutional environment (i.e., a bigger *Marketization Index*), the reduction effect of the pilot policy on residents' medical expenses becomes greater, thereby basically confirming **Hypothesis 2**.

**Table 8:** Mechanism analysis.

**Variables**	**(1) ln*ME_ outpatient***	**(2) ln*ME_ inpatient***	**(3) ln*ME_ outpatient***	**(4) ln*ME_ inpatient***
Policy	0.0990^**^	0.2008^***^	0.0723^***^	0.0094
	(0.0499)	(0.0442)	(0.0268)	(0.0244)
Competition	0.0497	−0.0388		
	(0.0636)	(0.0563)		
Policy × competition	−0.1513^*^	−0.3579^***^		
	(0.0793)	(0.0701)		
Market			0.0119^**^	0.0224^***^
			(0.0060)	(0.0055)
Policy × market			−0.0090^***^	−0.0047
			(0.0032)	(0.0029)
Control variables	Yes	Yes	Yes	Yes
Constant	Yes	Yes	Yes	Yes
Province FE	Yes	Yes	Yes	Yes
Year FE	Yes	Yes	Yes	Yes
*N*	270	270	270	270
*R^2^*	0.9712	0.9643	0.9721	0.9633

Standard errors are in parentheses. ^***^, ^**^, and ^*^ indicate significance at the 1, 5, and 10% levels, respectively.

### Heterogeneous analysis

#### Regional heterogeneity

**Figure 6:**
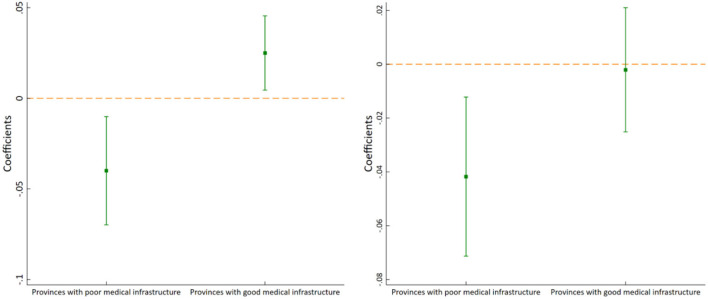
Regional heterogeneity of ln*ME_outpatient* **(left)** and ln*ME_inpatient* **(right)**.

According to different locations, the sample are divided into three sub-samples: eastern, central and western regions^1^. The estimated results of sub-samples are shown in Figure 6. As can be seen, whether ln*ME_outpatient* or ln*ME_inpatient* is the explained variable, the confidence interval (the vertical solid lines) of *Policy* covers value zero in the eastern and western regions, but not in the central region, indicating that the reduction effect of China's NCMR pilot policy on residents' medical expenses is significant in the central region but not in the eastern and western regions. The reason may be that the eastern region has a higher level of economic development, so that the residents there have a stronger awareness of health, and a higher ability and willingness to pay for medical expenses, which leads to the failure of the pilot policy to effectively reduce residents' medical expenses. On the contrary, in the western region, due to the low degree of marketization, the relevant reform is still dominated by the administration, and thus failing to effectively stimulate the reduction effect of the pilot policy on residents' medical expenses.

#### Medical infrastructure heterogeneity

**Figure 7:**
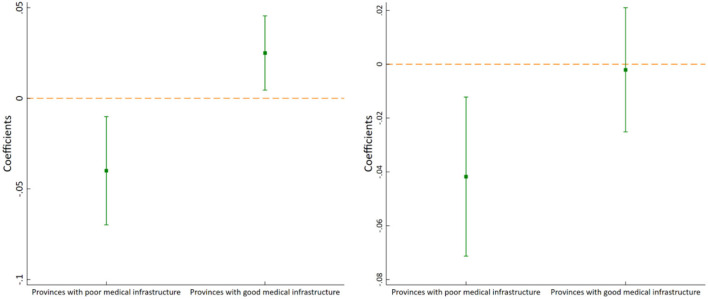
Medical infrastructure heterogeneity of ln*ME_outpatient* **(left)** and ln*ME_inpatient* **(right)**.

The medical infrastructure status is an important factor influencing the effectiveness of the pilot policy. For this reason, we divide the sample into two sub-samples based on the median of hospital beds per capita: provinces with poor medical infrastructure and provinces with good medical infrastructure. The regression results are shown in Figure 7. It can be seen that when ln*ME_outpatient* is used as the explained variable, the estimated coefficient of *Policy* is significantly negative in provinces with poor medical infrastructure, but significantly positive in provinces with good medical infrastructure, indicating that the pilot policy can effectively reduce medical expenses of outpatients in provinces with poor medical infrastructure, but increase medical expenses of outpatients in provinces with good medical infrastructure. When ln*ME_inpatient* is used as the explained variable, the estimated coefficient of *Policy* is significantly negative in provinces with poor medical infrastructure, but not significant in provinces with good medical infrastructure, which indicates that the pilot policy can effectively reduce medical expenses of inpatients in provinces with poor medical infrastructure, but has no significant effect on medical expenses of inpatients in provinces with good medical infrastructure. In brief, the reduction effect of the pilot policy on residents' medical expenses is significant only in provinces with poor medical infrastructure. The reason may be that the pilot policy can effectively improve access to medical services and reduce medical expenditure in provinces with poor medical infrastructure. This conclusion also indicates that China's NCMR pilot policy has the nature of “supporting the weak” to some extent.

#### Financial strength heterogeneity

**Figure 8:**
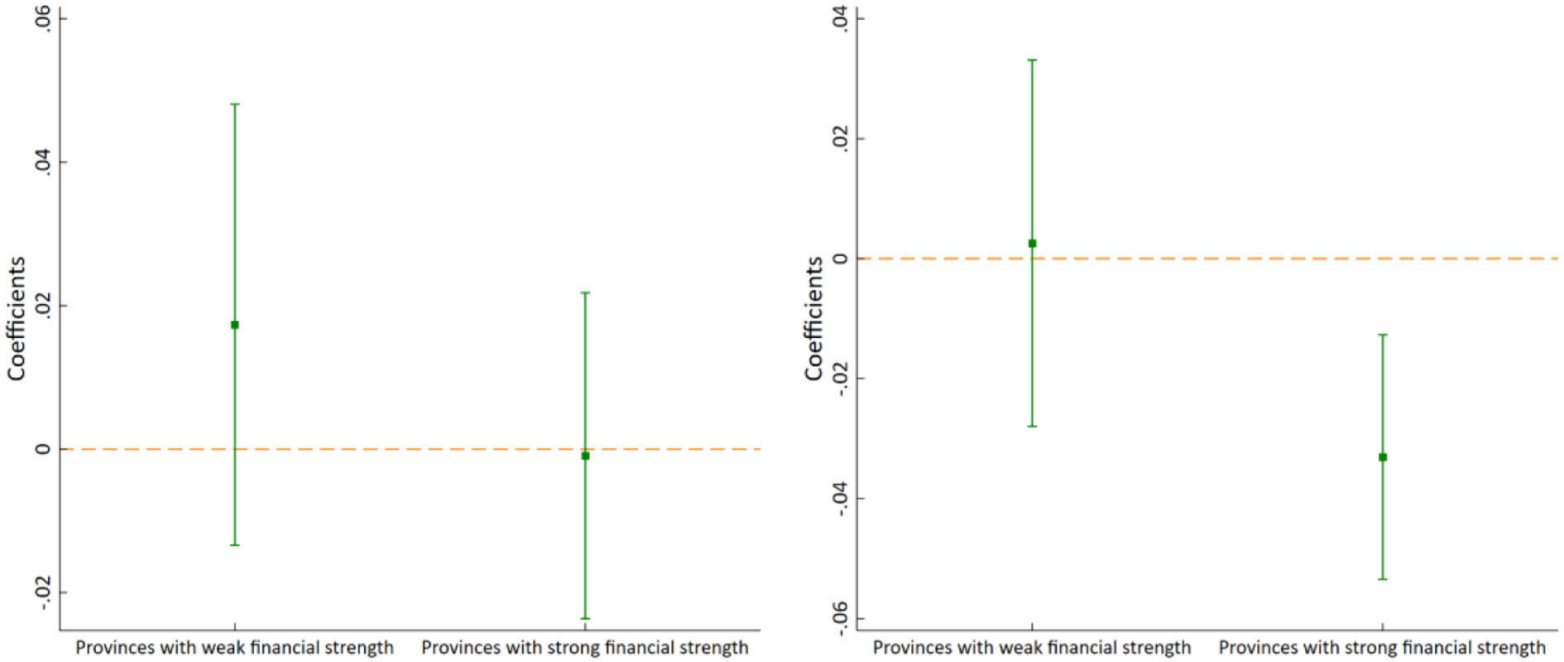
Financial strength heterogeneity of ln*ME_outpatient* **(left)** and ln*ME_inpatient* **(right)**.

Financial strength is an important guarantee for the effective implementation of medical reform. Therefore, we divide the sample into two sub-samples based on the median of general budgetary revenue: provinces with weak financial strength and provinces with strong financial strength. The estimation results are shown in Figure 8. It can be seen that when ln*ME_outpatient* is used as the explained variable, the estimated coefficient of *Policy* is not significant in provinces with both weak financial strength and strong financial strength. When ln*ME_inpatient* is used as the explained variable, the estimated coefficient of *Policy* is significantly negative in provinces with strong financial strength, but not significant in provinces with weak financial strength. Therefore, overall, the reduction effect of the pilot policy on residents' medical expenses is significant only in provinces with strong financial strength. The reason may be that provinces with stronger financial strength can invest more resources and funds into medical services, which can fully guarantee the effectiveness of the pilot policy.

## Discussion

High medical expense is not conducive to the improvement of residents' welfare and the realization of the common prosperity goal. Therefore, the influencing factors of residents' medical expenses are important topics that scholars have paid attention to for a long time. For example, the existing literature investigated the above issue from the perspective of air pollution (10–14), environmental regulation (15, 16), fiscal decentralization (17), and energy use (18).

As a new attempt of China's medical reform, the NCMR pilot policy also attracted the attention of scholars. However, there were few quantitative evaluations on the effect of the policy in the existing literature, and these studies mainly used the case study (9) or only focused on the policy effect of one province (28). In order to make up for the above shortcomings, this study uses the DID model to scientifically evaluate the impact of China's NCMR pilot policy on residents' medical expenses. In fact, effectively reducing residents' medical expenses is a challenge not only for China, but also for other countries, even developed countries. Our study provides new empirical evidence from the macro level of medical reform, which can provide a reference for governments to make decisions to some certain extent.

## Conclusion

Using the exogenous shock of China's NCMR pilot policy in 2015 and 2016, we construct a DID model to comprehensively investigate the impact and the mechanisms of the pilot policy on residents' medical expenses. The conclusions are as follows: first, the pilot policy effectively reduced residents' medical expenses, but mainly for inpatients other than outpatient patients. This research conclusion is still valid after a series of robustness tests such as the parallel trend test, the PSM-DID estimation, the entropy balancing estimation, the placebo test, controlling the time trend, and changing the sample interval. Second, hospital competition and institutional environment are important for the fully realizing of the policy effect. The more intense the hospital competition and the better the institutional environment, the greater the effect of the pilot policy on reducing residents' medical expenses. Third, the effect of the pilot policy on residents' medical expenses is heterogeneous. Specifically, the residents' medical expenses reduction effect is more effective in the central provinces, the provinces with poor medical infrastructure, and the provinces with strong financial strength.

Based on the above research conclusions, the following policy recommendations are proposed: first, we should further promote medical reform and expand the coverage of the pilot policy. In the horizontal dimension, further pilot provinces can be added on the basis of summarizing the successful experience of existing pilot provinces; in the vertical dimension, pilot policies can be tried to be implemented at the municipal or county level. Second, we should take relevant measures to support private health care institutions to provide basic health care services and strengthen hospital competition. At the same time, we should improve the regional institutional environment and promote the market-oriented reform of medical care to ensure the full play of the policy effect. Finally, in the further implementation of the pilot policy, it is important to tailor the policy to local conditions to ensure that the policy effects can be effectively implemented. Especially in those regions the policy effects have not been fully realized, relevant policy measures can be designed to adapt their actual situations.

Although this study is meaningful to understand the importance of medical reform in reducing residents' medical expenses, there are still some limitations. On the one hand, due to the availability of data, this study uses provincial panel data rather than city-level data or micro data of hospitals or individuals. Therefore, it is difficult for this study to effectively solve the problem of high sensitivity to costs in the field of medical management (42). On the other hand, due to different medical systems in different countries, the effectiveness of medical reform needs to vary according to the situation. This study mainly provides empirical evidence of China.

## Data availability statement

The original contributions presented in the study are included in the article/supplementary material, further inquiries can be directed to the corresponding author.

## Author contributions

CN was responsible for the overall design and writing of the article. YF was responsible for collecting data and establishing model. All authors contributed to the article and approved the submitted version.

## Footnotes

^1^The eastern region includes 11 provinces: Beijing, Tianjin, Hebei, Liaoning, Shanghai, Jiangsu, Zhejiang, Fujian, Shandong, Guangdong and Hainan; the central region includes eight provinces: Shanxi, Jilin, Heilongjiang, Anhui, Jiangxi, Henan, Hubei and Hunan; and the western region includes 11 provinces: Inner Mongolia, Guangxi, Chongqing, Sichuan, Guizhou, Yunnan, Shaanxi, Gansu, Qinghai, Ningxia and Xinjiang.
